# Caspase-1 participates in apoptosis of salivary glands in *Rhipicephalus haemaphysaloides*

**DOI:** 10.1186/s13071-017-2161-1

**Published:** 2017-05-08

**Authors:** Xinmao Yu, Yongzhi Zhou, Jie Cao, Houshuang Zhang, Haiyan Gong, Jinlin Zhou

**Affiliations:** 1 Key Laboratory of Animal Parasitology of Ministry of Agriculture, Shanghai Veterinary Research Institute, Chinese Academy of Agricultural Sciences, Shanghai, 200241 China; 2 Jiangsu Co-innovation Center for Prevention and Control of Important Animal Infectious Diseases and Zoonoses, Yangzhou, 225009 China

**Keywords:** Caspase-1, Apoptosis, Salivary glands, Autophagy

## Abstract

**Background:**

Ticks are among the most harmful vectors worldwide. Their salivary glands play essential roles in blood-feeding and pathogen transmission and undergo apoptosis after feeding. Although it was previously reported that salivary degeneration in ixodid ticks is in response to hormonal stimulation, questions still exist with the underlying mechanisms of salivary gland apoptosis.

**Methods:**

Salivary glands of *Rhipicephalus haemaphysaloides* were collected from 1 to 7 days after attachment to the host. TUNEL and Annexin V assays were used to check apoptosis during this time. To confirm the role of caspase-1, RNA interference was used to silence its expression, and the dynamic changes of associated cysteine proteases were also shown by quantitative real time PCR and western blot, while TUNEL and Annexin V assays were used to confirm apoptosis.

**Results:**

In the present study, apoptosis of salivary glands in *R. haemaphysaloides* occurred 3 or 4 days after attachment to the host as determined by TUNEL and Annexin V assays. The expression of caspase-1 increased at 5–7 days. When the latter was silenced by RNA interference, apoptosis in the salivary glands was delayed. While there seemed to be another form of cell death in salivary glands of ticks, such occurrence may be caused by compensatory autophagy which involved autophagy-related gene 4D.

**Conclusions:**

This study describes the apoptosis of salivary glands in *R. haemaphysaloides* and the dynamic changes in cysteine proteases in this activity. Cysteine proteases were involved in this process, especially caspase-1. Caspase-1 participated in the apoptosis of salivary glands.

**Electronic supplementary material:**

The online version of this article (doi:10.1186/s13071-017-2161-1) contains supplementary material, which is available to authorized users.

## Background

Ticks are the most harmful vectors after mosquitoes. They bite the host body and suck blood, leading to anaemia, and transmit numerous pathogens including bacteria, viruses and fungi during their feeding [1, 2]. Salivary glands play essential roles in blood-feeding and become vestigial after engorgement. The first report on this phenomenon was in 1986 in *Rhipicephalus sanguineus* [3]. Recent studies have reported that the changes in salivary glands were caused by programmed cell death [4]. In *Dermacentor variabilis* and *Boophilus microplus*, cell death was induced by apoptosis [5–8]. In addition, Scopinho Furquim et al. [9] showed that the apoptosis of salivary glands occurred in 2–3 days after feeding.

Apoptosis was first described by Kerr et al. [10], and it is usually activated through an endogenous or exogenous apoptosis pathway [11]. Caspases, which belong to the cysteine proteases, are among the main participants of apoptosis, which are mostly synthesized as proenzymes. In mammals, there are 12 caspase members, named caspase-1–12. Among them, caspase-8 and caspase-10 are the initiator caspases that are activated in the death-induced signalling complex (DISC) and induce activation of downstream caspases and the exogenous apoptosis pathway through interaction mediated by death effector domains [12]. Dimerisation of initiator caspases is promoted by multiprotein activation complexes (activated through an induced proximity mechanism), such as the apoptosome, DISC, and inflammasomes [11, 13]. The effector caspases (such as caspase-3, caspase-6 and caspase-7) are activated by specific initiator or upstream caspases in a cascade-like manner after cleavage [14]. Caspase-1 is a downstream caspase that is essential for the maturation of interleukin (IL)-1β and IL-18 [15]. IL-1β and IL-18 must be cleaved by caspase-1 and this is the first step for their maturation. Similar with other caspases, caspase-1 is also an inactive proenzyme until activated [16].

Autophagy is another pathway that leads to cell death; often accompanied by apoptosis [17]. Several types of proteases are involved in this activity [18]. Autophagy-related gene 4 (ATG4) is a type of cysteine protease that plays an important role in autophagy, especially for formation of ATG8 [19–24]. In arthropods, an autophagic process is involved in programmed cell death [25]. Autophagy has not been described in detail in ticks, although genes encoding ubiquitin have been reported in transcriptome analysis of tick salivary glands [26, 27]. Five homologues of autophagy-related genes, ATG3, ATG4, ATG6, ATG8 and ATG12, have been identified in the tick *Haemaphysalis longicornis*, which have distinct biological roles in eggs, unfed ticks and engorged ticks [28]. In *Rhipicephalus* (*Boophilus*) *microplus*, there were also five putative ATG genes, ATG3, ATG4, ATG6 and two ATG8s [29].

We have reported the salivary glands transcriptomes of *Rhipicephalus haemaphysaloides* and focused on the important cysteine proteases, including two caspases and ATG genes [30]. Ticks express cysteine peptidases with important roles in physiological events that are crucial to the ectoparasitic lifestyle, including digestion of host blood, embryogenesis and innate immunity [31]. However, there are few functional details about caspases or ATG genes in ticks.

In the present study, we confirmed apoptosis in salivary glands of *R. haemaphysaloides* by transferase-mediated deoxyuridine triphosphate-biotin nick end labeling (TUNEL) and Annexin V assay and identified caspase-1 and other cysteine proteases involved in this activity. We also found that interference with caspase-1 affected apoptosis, although apoptosis in salivary glands was not stopped. The apoptotic activity may be compensatorily regulated by autophagy that involves ATG4D to maintain salivary glands degradation.

## Methods

### Collection of ticks and salivary glands

*Rhipicephalus haemaphysaloides* colonies were maintained in the laboratory as described previously [32]. For tissue collection, the salivary glands were dissected and observed under a light microscope [32]. The sample materials were stored at -80 °C until use.

### TUNEL assay of salivary glands

*Rhipicephalus haemaphysaloides* adult ticks were fed on the ears of rabbits and collected at 24 h after biting. Salivary glands were dissected and processed by TUNEL assay kit (Roche, Welwyn Garden, UK). Salivary glands were fixed in 4% methanol-free formaldehyde for 20 min, embedded in paraffin, and cut into sections. The paraffin sections were washed in dimethylbenzene, graded ethanol and PBS several times, and permeated by cell permeation buffer at room temperature for 10 min. Before adding TUNEL reaction mix and the lid, and incubating for 1 h at 37 °C in a humidified atmosphere in the dark, the sections were washed in PBS several times and dried. After washing 3 times in PBS, the sections were blocked and observed under fluorescence microscopy.

### Annexin V-FITC assay of salivary glands

*Rhipicephalus haemaphysaloides* adult ticks were fed on the ears of rabbits and collected at 24 h after biting. Salivary glands were dissected and processed by Annexin V-FITC Apoptosis Detection Kit (Dojindo Laboratories, Tokyo, Japan). The salivary glands cells were centrifuged at 1,000× *rpm* for 3 min and the supernatant was removed. The cells were washed twice in PBS and 10-fold diluted Annexin V binding solution was added to make a final cell concentration of 10^6^ cells/ml. Before incubating the cells for 15 min at room temperature with protection from light, we added 5 μl Annexin V-FITC conjugate followed by 5 μl propidium iodide (PI) solution to the cell suspension. Finally, we added 400 μl 10-fold diluted Annexin V binding solution and subjected the solution to flow cytometry.

### RNA interference of caspase-1 in *R. haemaphysaloides* adult ticks

Caspase-1 double-stranded RNA (dsRNA) was synthesized using the T7 RiboMax™ Express Large Scale RNA Production System (Promega, Madison, WI, USA). The plasmid and primers have been described previously [30]. DsRNA (1 μg dsRNA per tick) was injected into unfed adult *R. haemaphysaloides* ticks by a micropipette puller. Control ticks were injected with an equal volume of PBS. Injected ticks were observed for 18 h before feeding on the rabbits, as described previously [33].

### TUNEL and Annexin V-FITC assays of salivary glands from caspase-1-silenced ticks

The ticks were collected at 24 h after biting and the salivary glands were dissected and observed under a light microscope as described above. TUNEL and Annexin V-FITC assays were also performed as described above.

### Relative expression analysis of cysteine proteases in salivary glands after RNA silencing

Total RNAs were purified from female tick salivary glands at different days after attachment. The cDNAs were synthesized from 200 ng RNA using random 6-mer primers with the PrimeScript RT Reagent Kit (Perfect Real Time) (TaKaRa, Shiga, Japan) using the following program: 37 °C for 15 min, 85 °C for 7 s, and finally 4 °C for hold. Quantitative real-time PCR was performed using SYBR Premix Ex Taq (TaKaRa) with a StepOnePlus Real-Time PCR System (Applied Biosystems, New York, USA), with cycling parameters of 95 °C for 30 s, followed by 40 cycles of 95 °C for 5 s and 60 °C for 30 s. All the sequences and primers were based on our previous study [30]. Gene-specific standards were the respective plasmids. All samples were analysed three times.

The data were normalized to the elongation factor-1 gene (EF-1) (accession number AB836665) [34]. Relative gene expression data were analysed using the 2^-ΔΔCt^ method [35, 36], and ΔCt values were calculated by subtracting the average EF-1 Ct values from those for the average target gene.

### Western blot analysis of cysteine proteases in salivary glands from caspase-1-silenced ticks

Salivary glands (collected from RNAi and control group female ticks) were ground with PBS and centrifuged at 8000× *g* for 10 min at 4 °C. The supernatants were collected. The protein extracts from salivary glands were subjected to 10% SDS-Tris-Tricine gel electrophoresis and then transferred onto nitrocellulose membranes. The membranes were blocked with 5% non-fat milk for 2 h at 37 °C and washed 5 times with PBS. The antibodies were diluted in PBS-0.1% Tween 20 and used in the incubation steps as follows: polyclonal antibody (primary antibody serum form mice, dilution 1:100, at 4 °C, overnight) and HRP-conjugated goat anti-mouse IgG (secondary antibody, dilution 1:4,000, for 1 h at room temperature). The immunoreactive bands were detected by Tanon 2500 Gel Imaging System (Tanon, Shanghai, China). Based on our previous study [30], purified recombinant proteins (Cathepsin L and B (CATL and CATLB), Caspase-1(CASP1), ATG4B and ATG4D) were injected into mice at 2-week intervals. The hyper-immune sera were collected for primary antibodies.

### Statistical analysis

Significant difference in results of “TUNEL and Annexin V-FITC assays of salivary glands from caspase-1-silenced ticks” and “Relative expression analysis of cysteine proteases in salivary glands from caspase-1-silenced ticks by quantitative real-time PCR” were determined by two-tailed Student’s *t*-test using GraphPad PRISM 5.0 software (La Jolla, CA, USA). Statistical significance threshold was set as α = 0.05 and assigned at the level of *P* < 0.05.

## Results

### Anatomical observation of salivary glands

**Fig. 1:**
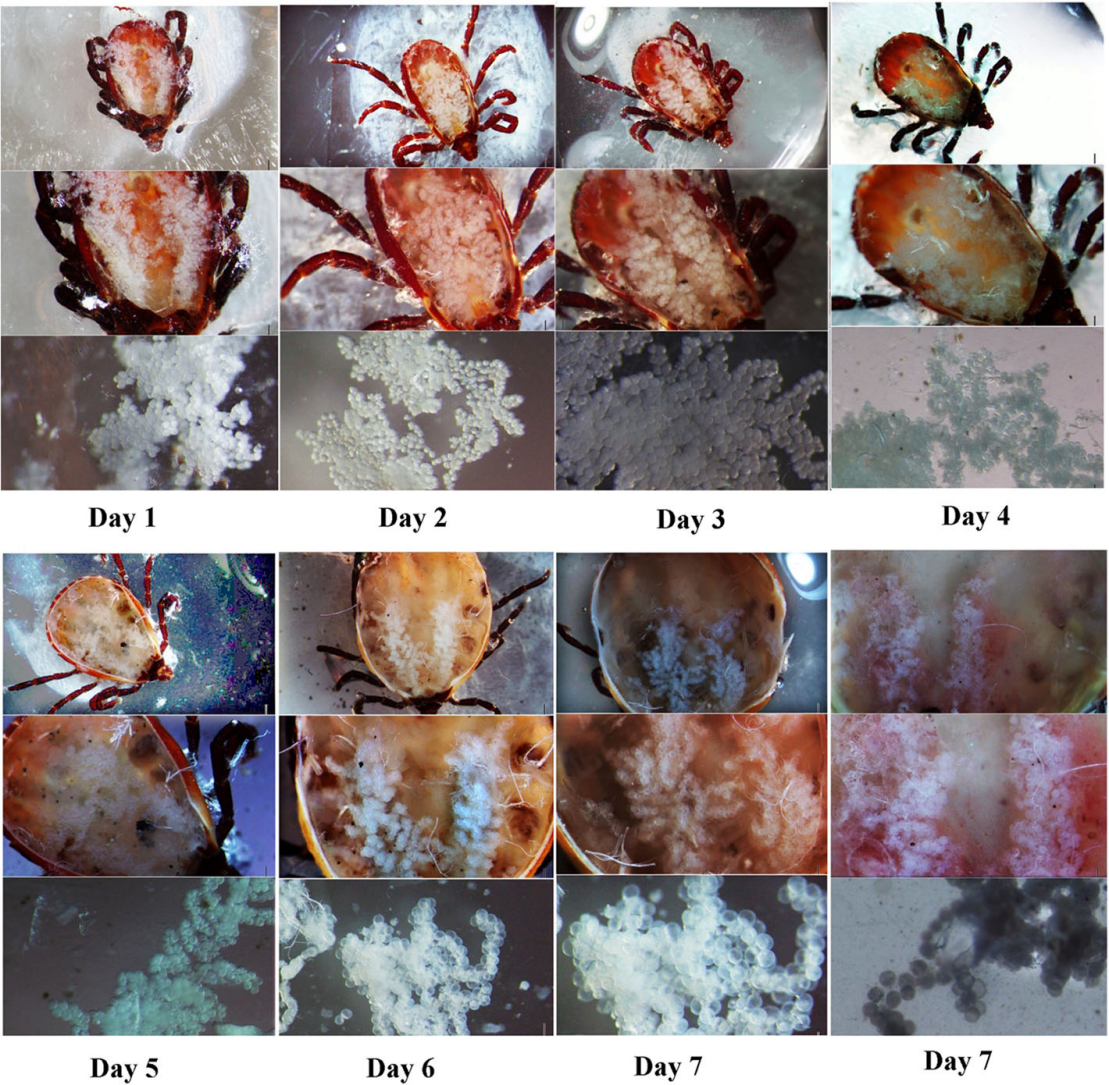
*Rhipicephalus haemaphysaloides* salivary glands in different days after attachment. First line: low-power field of salivary glands in ticks; second line: high-power field of salivary glands in ticks; third line: high-power field of salivary glands in vitro

*Rhipicephalus haemaphysaloides* adult female ticks were engorged in about 7 days. The salivary glands were collected every day and observed under a light microscope (Fig. 1). The salivary glands in 2 or 3 days were plump and distinct, while in 4 or 5 days, they became withered and indistinct. After 7 days, the lineament of the salivary glands was blurred and clearly dispersed.

### TUNEL assay for salivary glands

**Fig. 2:**
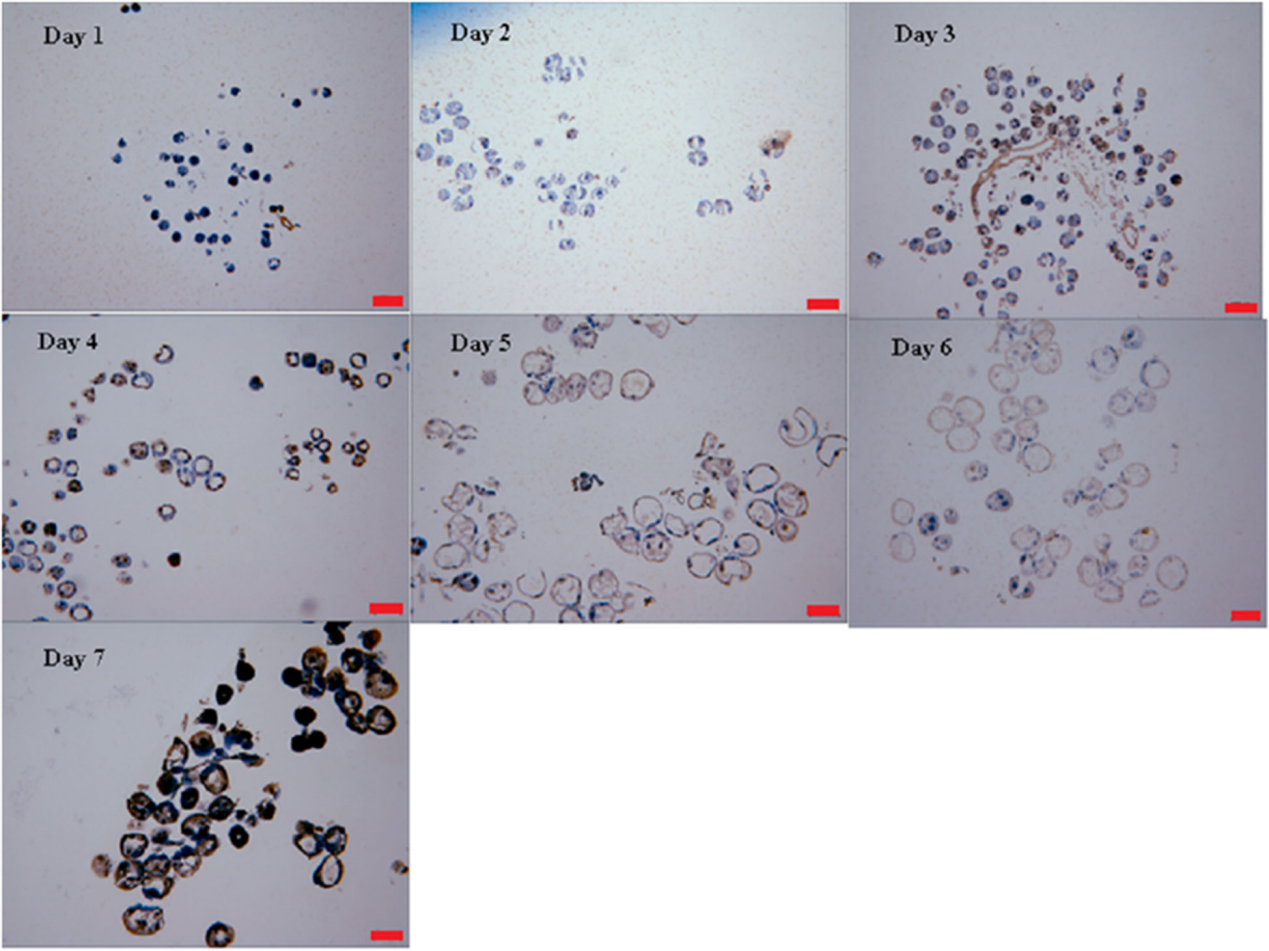
TUNEL assay for the different days of *Rhipicephalus haemaphysaloides* salivary glands. *Scale-bars*: 100 μm

The salivary glands were collected every day and subjected to TUNEL assay (Fig. 2). On days 1 and 2, almost all the salivary glands were normal. Apoptosis in the salivary glands occurred at 3 and 4 days after attachment. At day 5 and 6, the salivary glands were degraded. At day 7, the ticks were engorged and all salivary glands were necrotic or degraded and non-functional.

### Annexin V-FITC assay of salivary glands

**Fig. 3:**
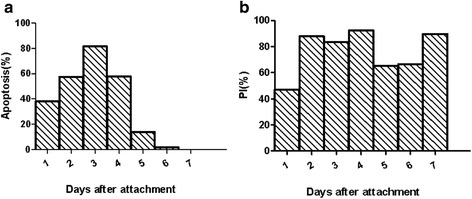
Apoptosis at different days after attachment. **a** Proportion of apoptotic cells detected by Annexin V. **b** Proportion of dead cells detected by PI

The salivary glands were processed and subjected to flow cytometry and results is shown in Fig. 3. During the first 3 days, the apoptosis ratio was increasing and at day 3, the ratio was highest at 81.5% (Fig. 3a). At 4 days, the proportion of dead cells was highest at 92% (Fig. 3b).

### TUNEL and Annexin V-FITC assays of salivary glands from caspase-1-silenced ticks

**Fig. 4:**
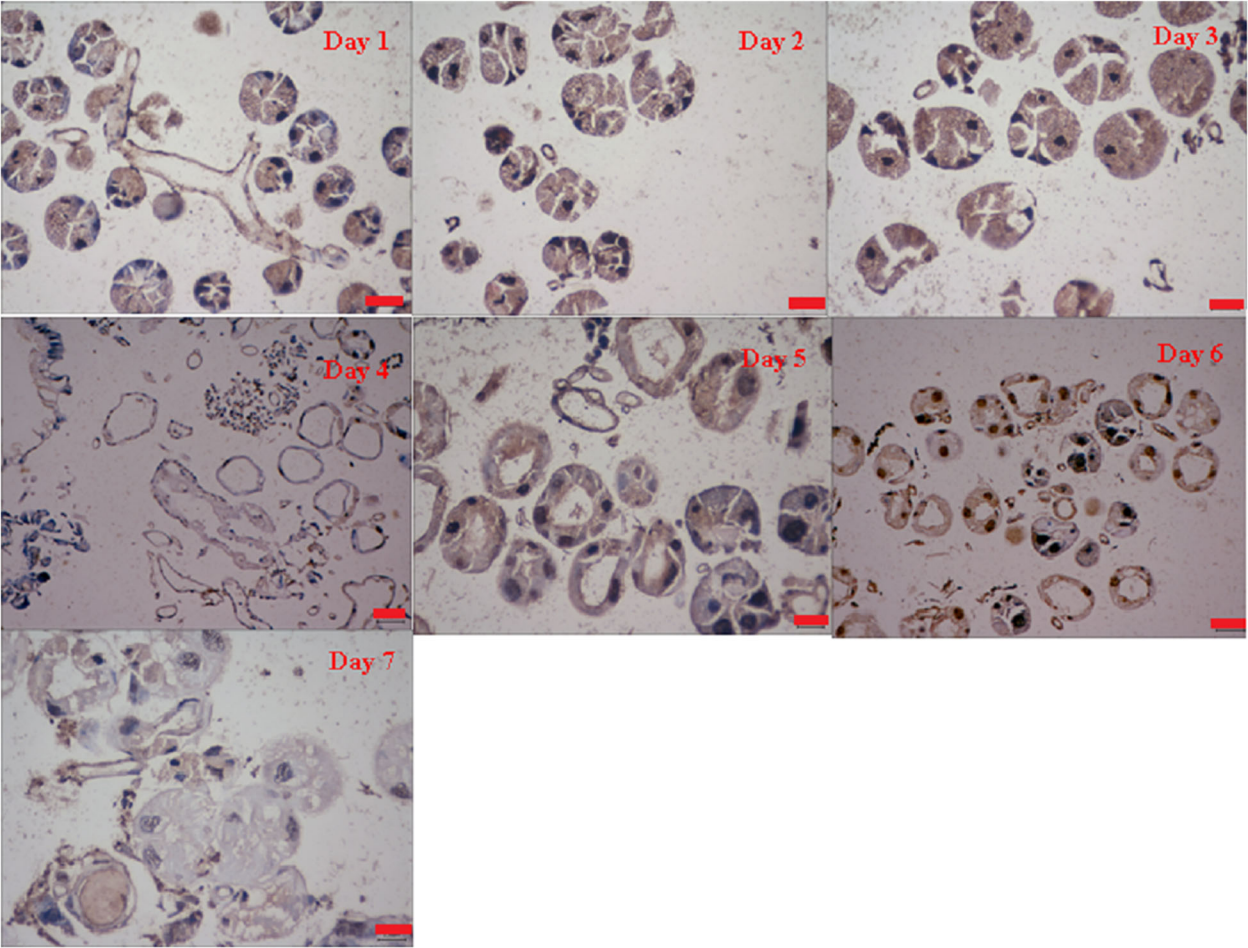
CASP1-RNAi in *R. haemaphysaloides* salivary glands at 7 days after attachment, as shown by TUNEL assay. *Scale-bar*: 25 μm

**Fig. 5:**
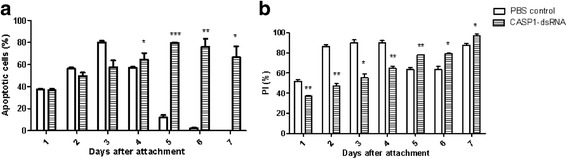
Apoptosis of salivary glands of CASP1-silenced ticks on different days after attachment. **a** apoptotic cells detected by Annexin V. **b** Dead cells detected by PI. Each experiment was repeated three times. Significant difference analysis of mean values was compared by *t*-test. *P < 0.05; **P < 0.01; ***P < 0.001

Adult female ticks were injected with dsRNA-caspase-1, and their salivary glands were collected each day after attachment. The results of TUNEL and Annexin V-PI assays are shown in Figs. 4 and 5, respectively. Apoptosis was identified by TUNEL assay after 3 and 4 days; at 5 and 6 days, the salivary glands cells were degraded; and at 7 days, almost all cells were dead (Fig. 2). When caspase-1 was silenced by specify dsRNA (CASP-1 dsRNA), apoptosis was delayed. During the first 4 days, the morphology of the salivary glands was not as clear as usual (Fig. 4). At days 5 and 6, apoptosis in the salivary glands was obvious, and at day 7, the CASP-1-silenced group salivary glands cells were blurred and degraded, which differed from the control group. Analysis of Annexin V also showed that apoptosis was deferred significantly (Fig. 5). The proportion of apoptotic cells increased significantly on days 5–7 (Fig. 5a) (day 4: *t*_(4)_ = 2.4874, *P* = 0.04974; day 5: *t*_(4)_ = 27.338, *P* < 0.001; day 6: *t*_(4)_ = 17.863, *P* < 0.01; day 7: *t*_(4)_ = 10.004, *P* < 0.001). The proportion of dead cells was reduced in the first 4 days, but increased significantly in the last 3 days (Fig. 5b) (day 1: *t*_(4)_ = 7.9046, *P* < 0.01; day 2: *t*_(4)_ = 10.982, *P* < 0.001; day 3: *t*_(4)_ = 9.621, *P* < 0.001; day 4: *t*_(4)_ = 11.165, *P* < 0.001; day 5: *t*_(4)_ = 4.0334, *P* = 0.01569; day 6: *t*_(4)_ = 4.4056, *P* = 0.01164; day 7: *t*_(4)_ = 2.776, *P* = 0.05745).

### Dynamic changes of cysteine proteases transcription in salivary glands from caspase-1-silenced ticks

**Fig. 6:**
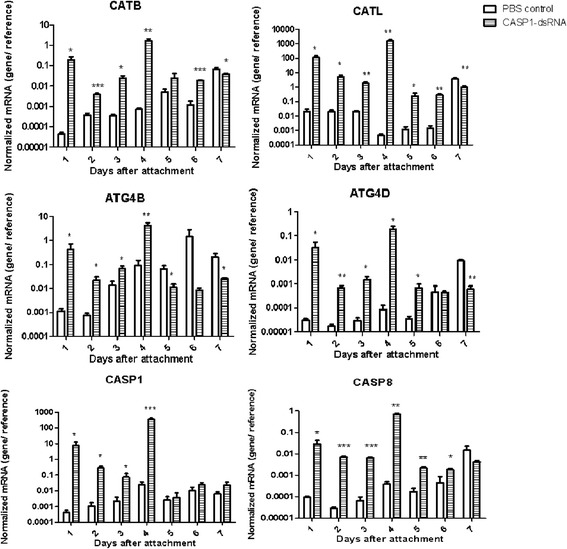
Dynamic changes of cysteine proteases transcription in adult female tick salivary glands of *R. haemaphysaloides* by CASP1-RNAi during seven days after attachment. ELF IA gene served as the endogenous control. PBS served as the control group. Each experiment was repeated three times. Significant differences in *t*-tests are indicated. *P < 0.05; **P < 0.01; ***P < 0.001

The salivary glands were collected every day and total RNA was extracted for reverse transcription. The experimental group was injected with dsRNA-CASP1, with an equal volume of PBS in the control group. According to our previous study [30], six cysteine proteases were detected by real-time PCR (Fig. 6). All cysteine protease transcriptions were upregulated significantly, especially at 4 days after attachment (CATB: day 4, *t*_(5)_ = 4.1926, *P* < 0.01; CATL: day 4, *t*_(5)_ = 4.303, *P* < 0.01; ATG4B: day 4, *t*_(5)_ = 3.8005, *P* < 0.01; ATG4D: day 4, *t*_(5)_ = 2.8427, *P* = 0.03849; CASP1: day 4, *t*_(5)_ = 5.9256, *P* < 0.001, CASP8: day 4, *t*_(5)_ = 5.9513, *P* < 0.01).

### Western blot analysis of cysteine proteases in salivary glands from caspase-1-silenced ticks

**Fig. 7:**
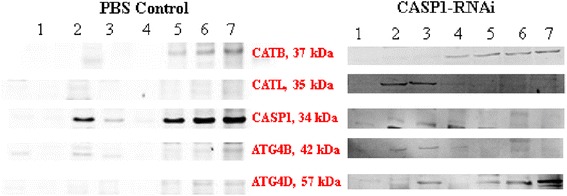
Cysteine proteases western blot of *R. haemaphysaloides* salivary glands during seven days after attachment. Lanes 1–7: different days of western blotting for cysteine proteases

Changes in cysteine protease expression were detected by western blotting (Fig. 7). Normally, the expression of caspase-1 was increased from days 5–7, while after being interfered, its expression was inhibited by CASP-1 dsRNA and there was little expression (silencing efficiency was over 95%) during days 5–7 When CASP1 was silenced, CATL was expressed on days 2 and 3. From days 5–7, expression of ATG4D was obviously increased.

## Discussion

The change in salivary glands in ticks seems to be a common phenomenon. In *D. variabilis* and *B. microplus*, cell death is induced by apoptosis [5–8, 37, 38]. Our study showed that the apoptosis in salivary glands occurred at 3–4 days after attachment, which differs from that in *R. sanguineus* [9]. To the best of our knowledge, this is the first report of apoptosis in *R. haemaphysaloides*. The salivary glands are a pathway for blood and pathogens and a guarantee for blood-feeding [39–41]. When ticks bite their host, their salivary glands become active immediately [42]. Numerous proteins are synthesized and secreted to resist the host immune response [40, 43, 44]. A balance is established between ticks and their host, so that the blood flows continuously in the future [45, 46]. That may explain why there is little blood in ticks in the first 3 days. During days 3–7, all the preparation for blood flow had been finished, and blood entered the gut rapidly through the salivary glands. During this rapid blood-sucking stage, the salivary glands seemed to form the only connection between the host and tick gut, and their main task had been completed. Salivary glands are regarded as a “useless” organ to be degraded by apoptosis, which is the ultimate fate of the salivary glands.

Caspases, as members of the cysteine protease family, are major participants in apoptosis. There are 12 caspases in humans, 11 in rodents (with loss of caspase-10), and 7 in *Drosophila* [12, 47, 48]. Two caspases are found in *H. longicornis* [49]. In *R. haemaphysaloides*, we confirmed the sequences of caspase-1 and caspase-8, and other caspases (such as caspase-3 and caspase-7) are detected in sialotranscriptomes [30]. Unfortunately, for caspase-3, caspase-7 and caspase-8, we did not get their recombinant protein and specific antibody, so we could not confirm the RNA interference effect in protein level. Although silencing caspase-3, caspase-7 and caspase-8 in transcriptional level did not produce promising results, it is more than likely that some regulatory mechanisms which involve these genes remain to be discovered. With such results, we decided to focus on caspase-1, but further research on caspase-3, caspase-7 and caspase-8 are still in progress.

Caspase-1 is regarded as an effector caspase and is responsible for production of IL-1β and IL-18 [15, 50]. Ticks only have an innate immune system and the function of caspase-1 is still a mystery. After biting the host, apoptosis began 2 or 3 days later, and expression of caspase-1 increased from 5 to 7 days. It seemed that caspase-1 was still acting as a downstream caspase to participate in salivary gland apoptosis. When caspase-1 was silenced by CASP-1 dsRNA, apoptosis was affected significantly and delayed, indicating the important role of caspase-1 in this activity. It is particularly interesting that although there were no cytokines synthesized by the ticks themselves, there must have been some in the host blood flowing into the tick gut. It still needs further study to confirm whether tick caspase-1 was involved in the production of these cytokines (e.g. IL-1β and IL-18).

The autophagy pathway is regarded as a protective mechanism. Cellular contents are transported to lysosomes for further digestion [51]. In *Saccharomyces cerevisiae*, there are several ATG genes and more than 30 ATG coding genes are found in yeast [52, 53]. In higher animals, there are also some ATG homologues that have been identified and characterised for autophagic roles [54]. Proteases are involved in different steps of autophagy and play important roles. In the initial stage of autophagy, ATG8 and conjugated phospholipid acyl amine participate in the formation of autophagic vacuoles [55]. In yeast, ATG4, which belongs to the cysteine proteases, cleaves part of the C terminus of ATG8, and then the processed ATG8 (ATG8^G116^) is involved in ubiquitin-like conjugation catalysed by ATG7 and ATG3 [55]. This is an essential step for autophagosome formation. And in mammalian cells, similarly, ATG4 is involved in the production of three ATG8 homologues, GABARAP, LC3 and GATE-16 [56, 57], which is also important for autophagy.

In *R. haemaphysaloides*, we confirmed 2 ATG sequences and renamed them as ATG4B and ATG4D based on BLAST and structural domain analysis in NCBI databases [30]. Although there are no details about the autophagic activity in *R. haemaphysaloides* to date, our research indicated that the autophagy is involved in the apoptosis of salivary glands, in cooperation with apoptosis induced by caspases. In our sialotranscriptome databases, there were some other predicted autophagy-related proteins or homologues, such as ATG2B, ATG3, ATG5, ATG7, ATG9A, ATG10, ATG12, ATG13, ATG14 and Beclin-1 [30]. Their functional details merit further exploration.

Autophagy and caspases are closely related with cell death. In *Drosophila*, caspases and ATG genes are expressed in dying larval salivary glands, suggesting that caspases and autophagy work synergistically for this cell death [58, 59]. Furthermore, inhibiting caspases or autophagic proteases only partially inhibits salivary gland degradation, and combined inhibition of caspases and autophagic proteases results in greater inhibition of salivary gland degradation [60, 61]. Recent studies have suggested that the relationship between these two activities may be parallel in dying cells in salivary glands. Decreased caspase function fails to inhibit autophagic activity, and ATG mutations neither inhibit caspase activity nor lead to premature caspase activity [61, 62]. In our study, caspases seemed to be complementarily regulated by autophagy. By day 7 TUNEL, the degradation of salivary glands had still been done, but in different form, and this was most probably caused by exchange of the cell death model from apoptosis to autophagy. These two activities must be involved in the degradation of salivary glands, while we consider that apoptosis induced by caspases is dominant. When it is interfered, autophagy is increased to maintain stability for salivary glands apoptosis.

To the best of our knowledge, this is the first study of *R. haemaphysaloides* to describe in detail the apoptosis of its salivary glands and reveal the relationship between caspase-1 and ATG4. We have previously reported the sialotranscriptomes of *R. haemaphysaloides* and focused on the function of cysteine proteases [30]. For apoptosis and autophagy, there are many other essential proteins in our databases and further studies are in progress.

## Conclusion

This is the first report that confirmed apoptosis in *R. haemaphysaloides* salivary glands which mainly occurred at 3–4 days after attachment. Cysteine proteases were involved in this process, especially caspase-1. Caspase-1 participated in the apoptosis of salivary glands and its expression increased at 5–7 days. When caspase-1 was silenced by dsRNA, the expression of ATG4 was increased and its degraded form in salivary glands was changed, suggesting that autophagy may be compensatorily regulated in salivary gland degradation.

## Additional files

Additional file 1: Annexin V-PI raw data file. (DOCX 1280 kb)

Additional file 2: Western blot RAW image. (PPT 1494 kb)

Additional file 3: Q-PCR raw data files. (RAR 51 kb)

## Abbreviations

ATG: Autophagy-related gene
CASP: Caspase
CAT: Cathepsin
DISC: Death-induced signalling complex
EF-1: Elongation factor-1 gene
GABARAP: Gamma-aminobutyric acid receptor-associated protein
IL: Interleukin
LC3: Light chain 3
PBS: Phosphate Buffered Saline
PI: Propidium iodide
TUNEL: Transferase-mediated deoxyuridine triphosphate-biotin nick end labeling
