# Supplementary material for: Caspase-1 participates in apoptosis of salivary glands in Rhipicephalus haemaphysaloides
Source: Parasit Vectors. 2017 May 8;10:225. doi: 10.1186/s13071-017-2161-1 (PMC5422879; doi:10.1186/s13071-017-2161-1)

PI-Annexin V RAW data

BD，FACSCalibur

PI-Annexin V Kit（Dojindo Laboratories, Tokyo, Japan）

Day 1: PI=47.05


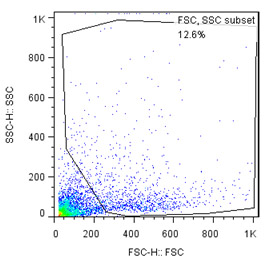

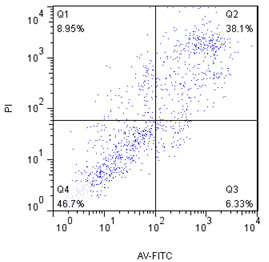


Day2: PI=87.7


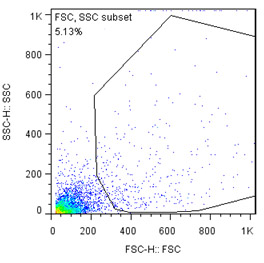

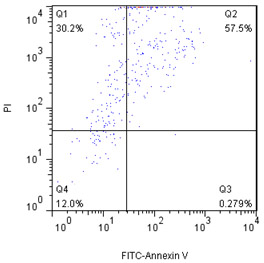


Day3: PI=83.65


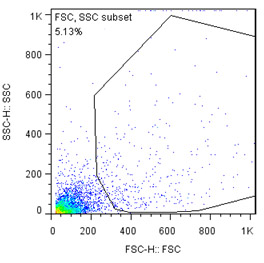

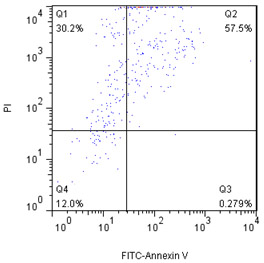


Day4: PI=92.2


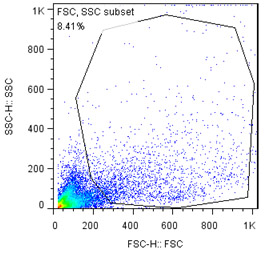

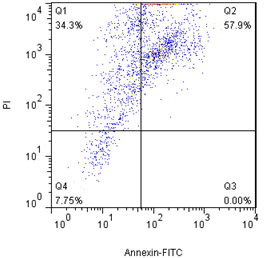


Day5: PI=65.4


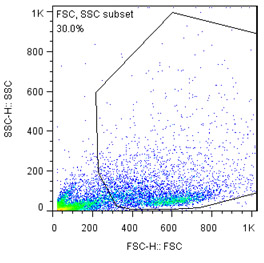

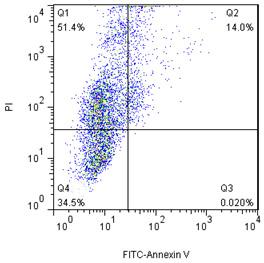


Day6: PI=66.36


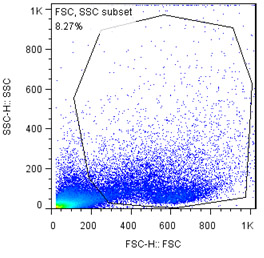

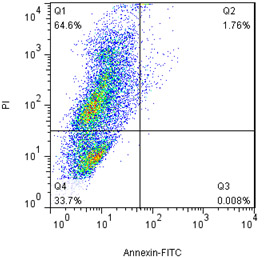


Day7: PI=89.562


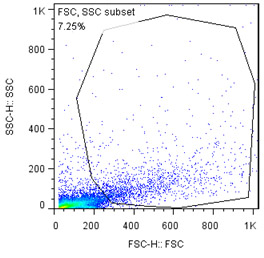

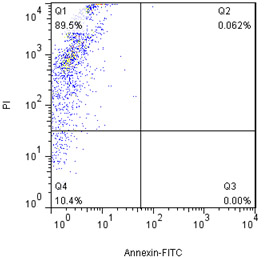


RNAi: PI-Annexin V assay:

Day 1:


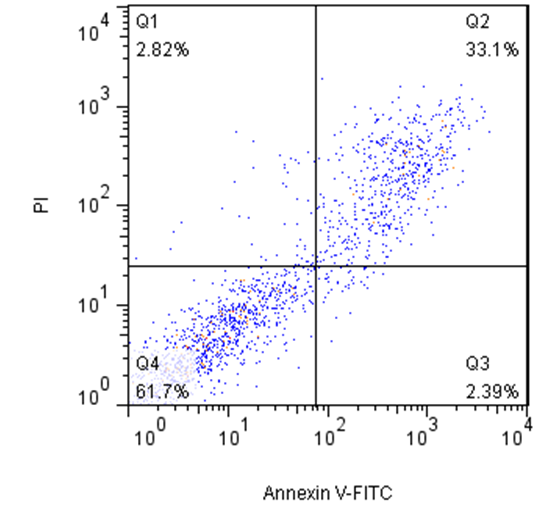

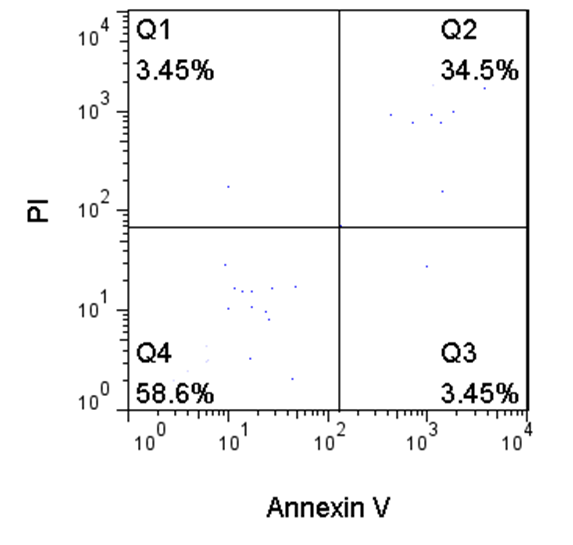


Day2:


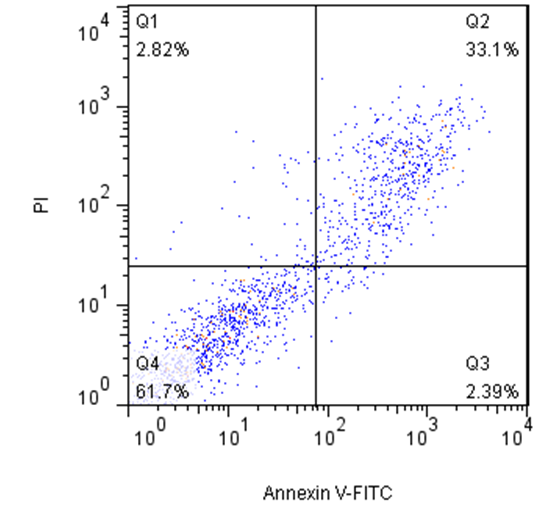

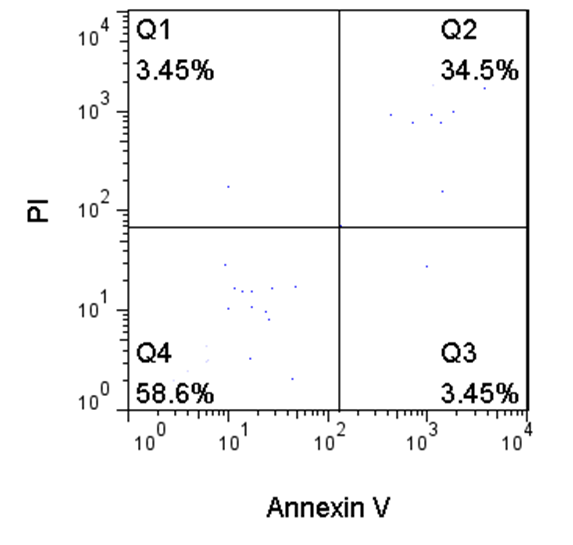


Day 3:


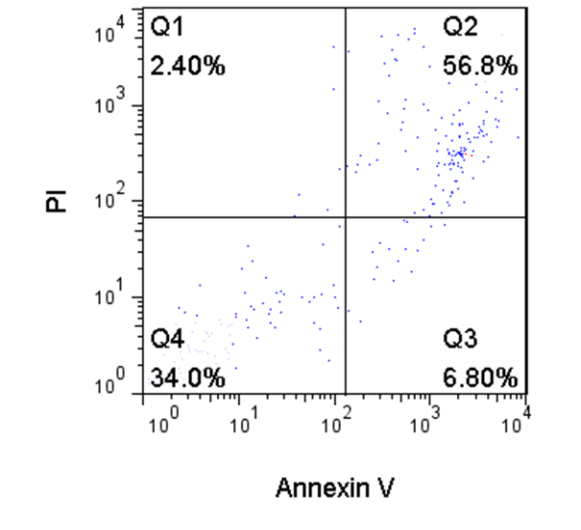

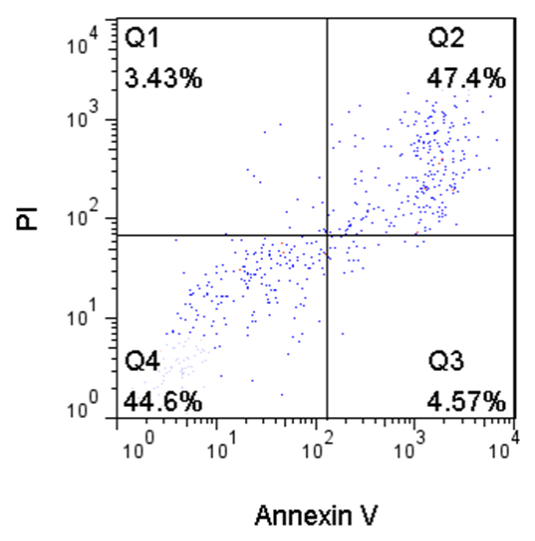


Day4:


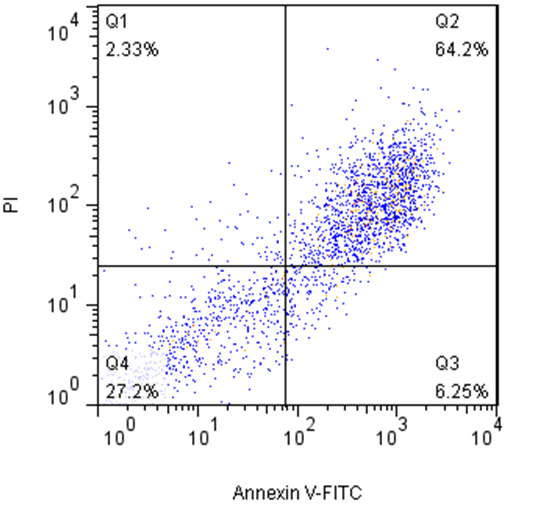

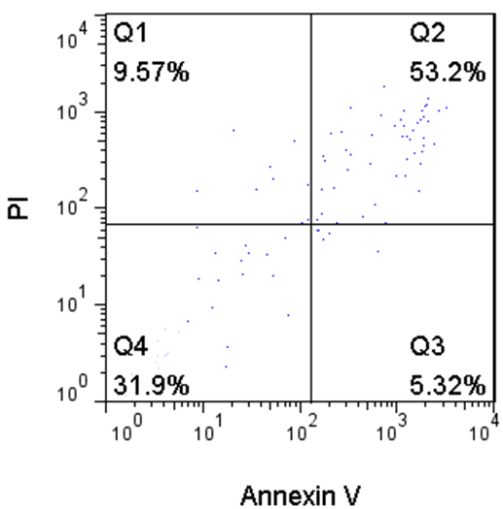


Day5:


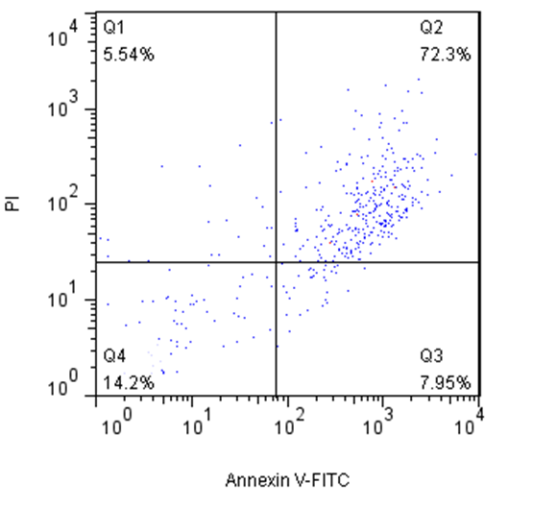

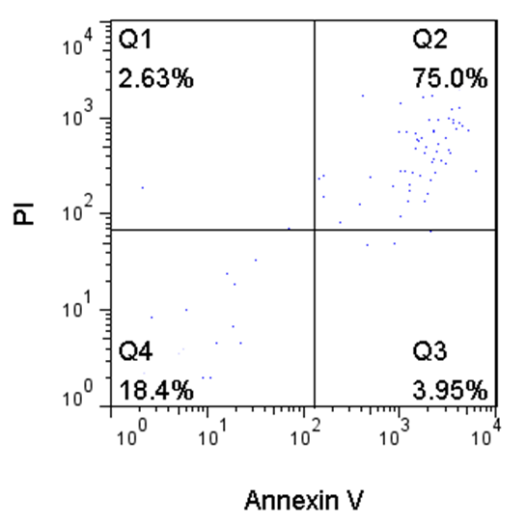


Day6:


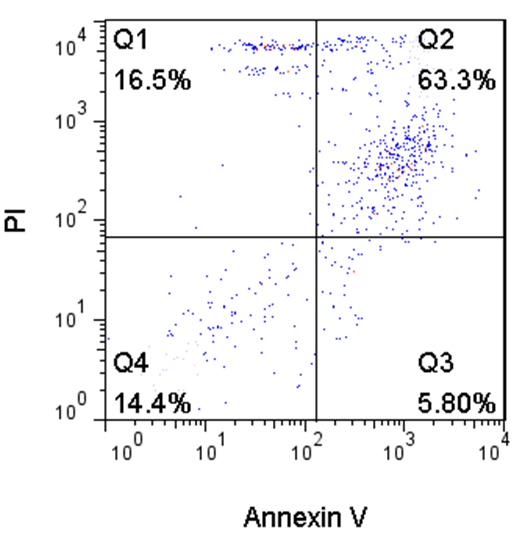

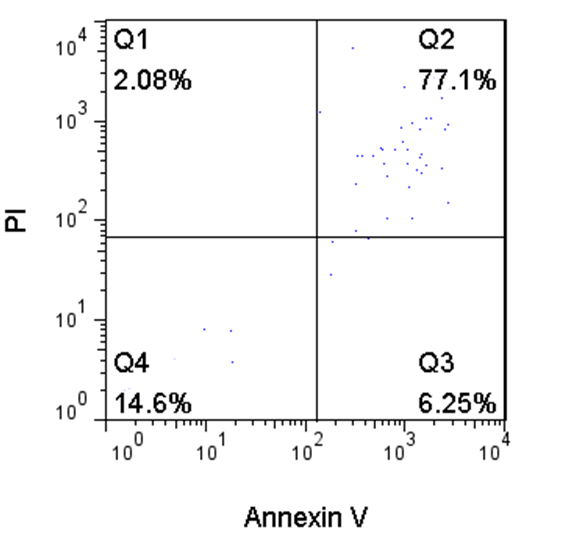


Day7:


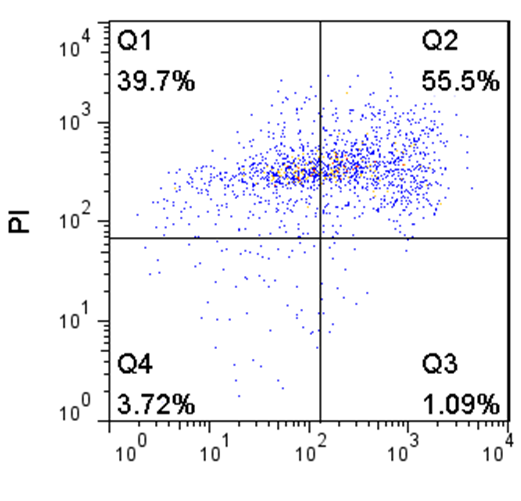

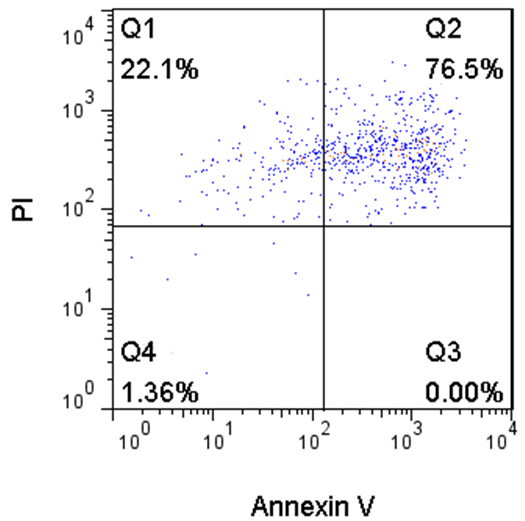

Supplement: Supplementary file 1 — Annexin V-PI raw data file. (DOCX 1280 kb) [file 13071_2017_2161_MOESM1_ESM.docx]
