# Supplementary material for: Caspase-1 participates in apoptosis of salivary glands in Rhipicephalus haemaphysaloides
Source: Parasit Vectors. 2017 May 8;10:225. doi: 10.1186/s13071-017-2161-1 (PMC5422879; doi:10.1186/s13071-017-2161-1)
Supplement: Supplementary file 2 — Western blot RAW image. (PPT 1494 kb) [file 13071_2017_2161_MOESM2_ESM.ppt]

## Slide 1
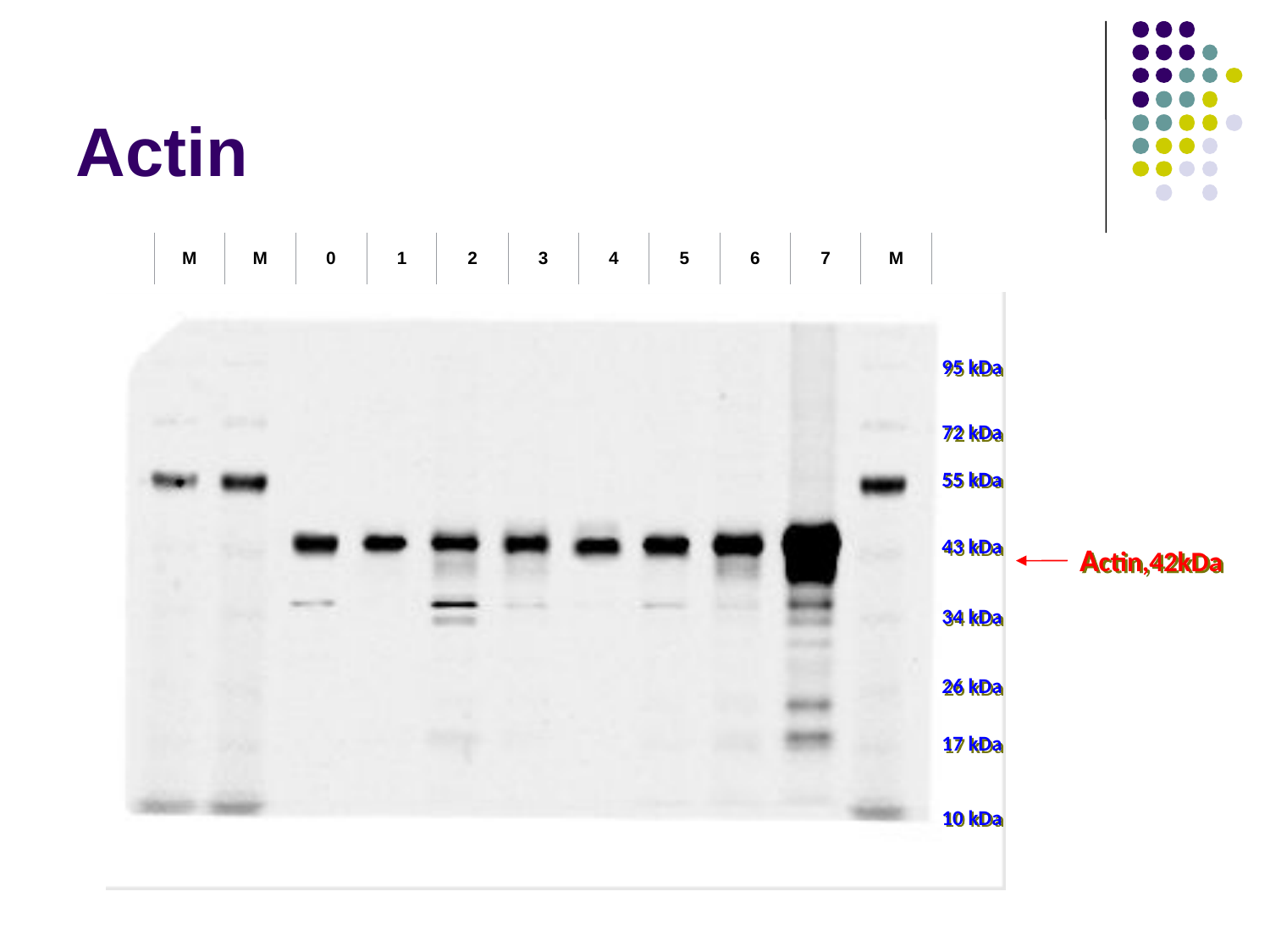

# Actin
| M | M | 0 | 1 | 2 | 3 | 4 | 5 | 6 | 7 | M |
| --- | --- | --- | --- | --- | --- | --- | --- | --- | --- | --- |
95 kDa
72 kDa
55 kDa
43 kDa
Actin,42kDa
34 kDa
26 kDa
17 kDa
10 kDa

## Slide 2
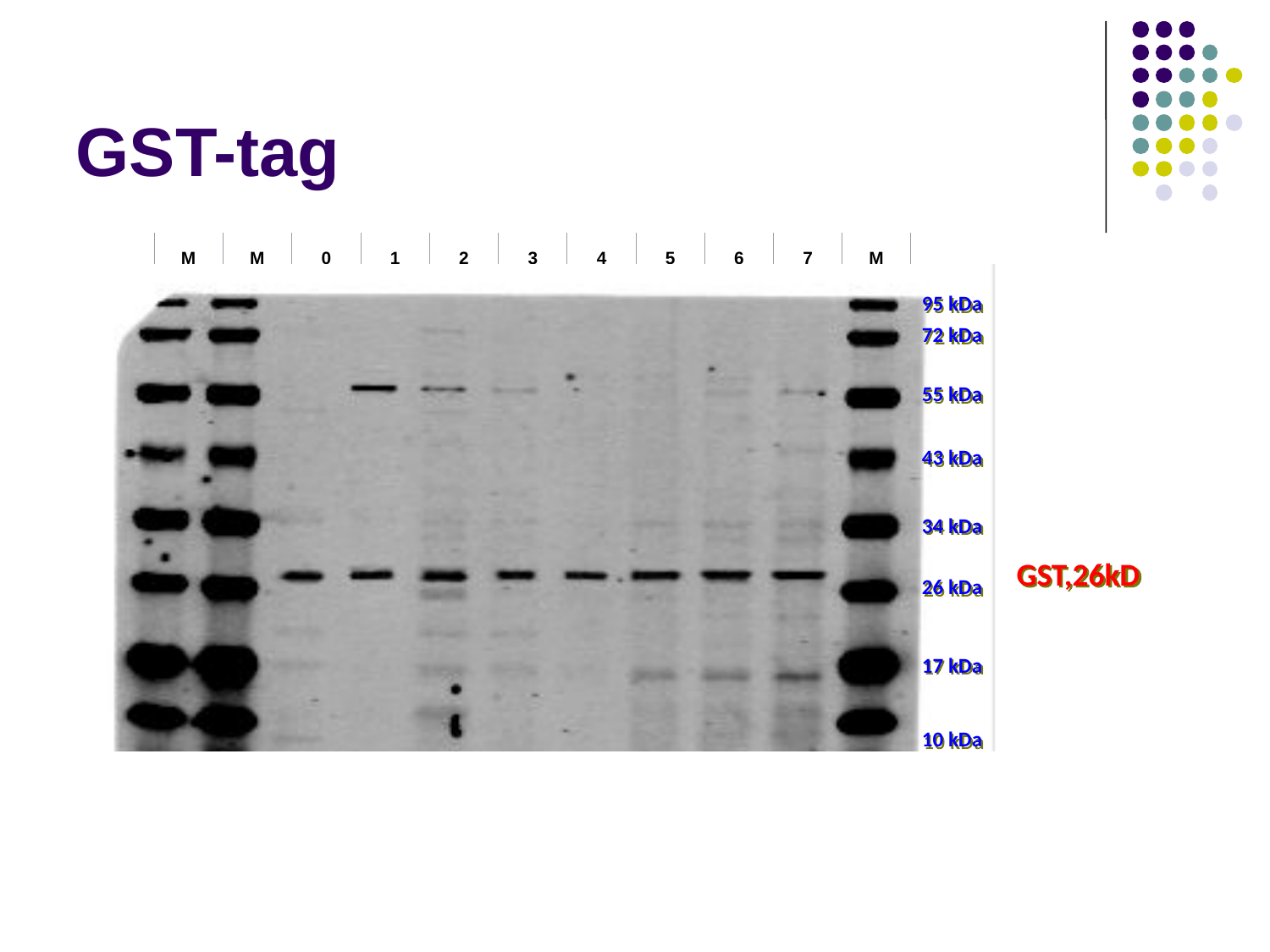

# GST-tag
| M | M | 0 | 1 | 2 | 3 | 4 | 5 | 6 | 7 | M |
| --- | --- | --- | --- | --- | --- | --- | --- | --- | --- | --- |
95 kDa
72 kDa
55 kDa
43 kDa
34 kDa
GST,26kD
26 kDa
17 kDa
10 kDa

## Slide 3
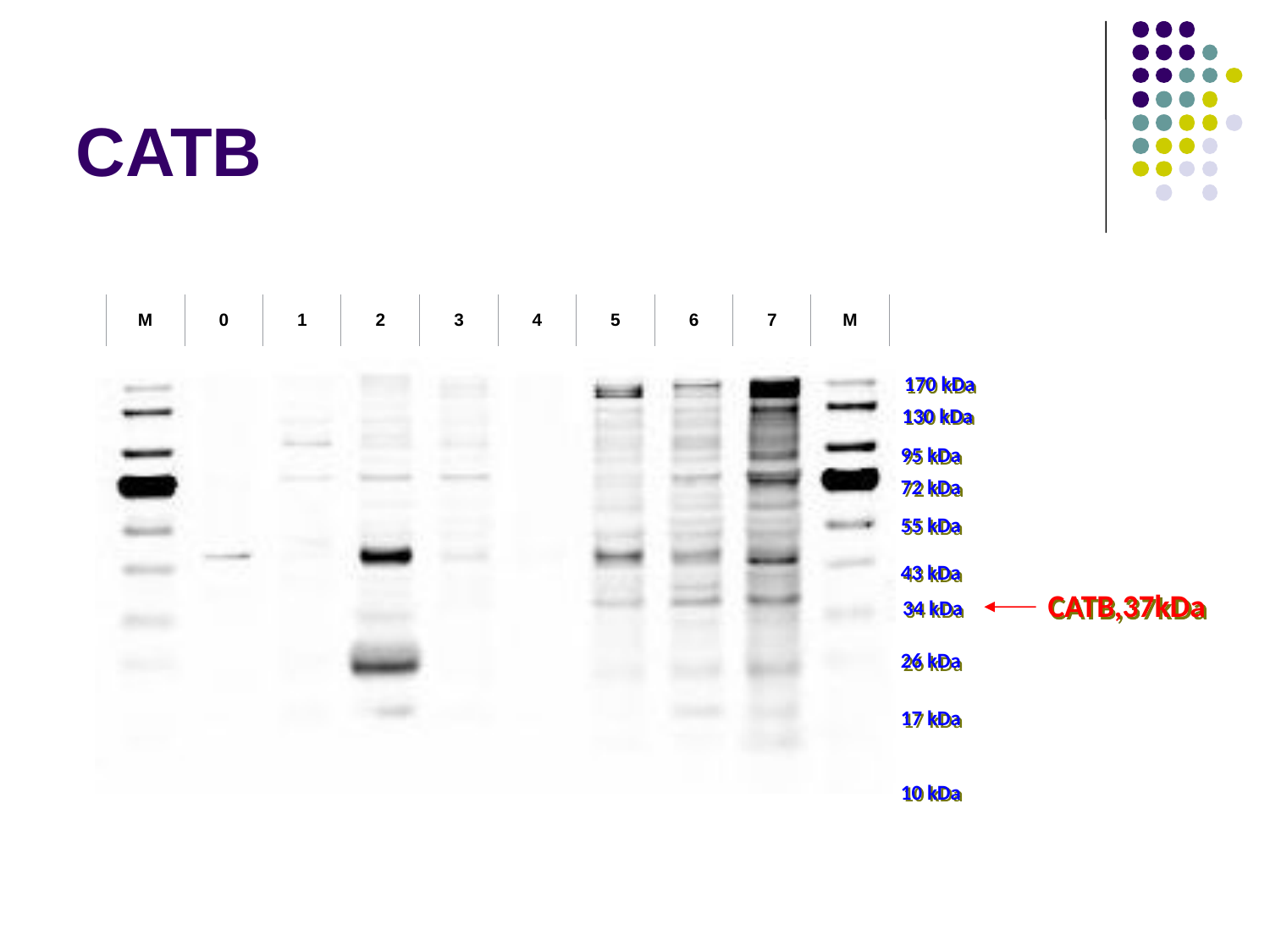

# CATB
| M | 0 | 1 | 2 | 3 | 4 | 5 | 6 | 7 | M |
| --- | --- | --- | --- | --- | --- | --- | --- | --- | --- |
170 kDa
130 kDa
95 kDa
72 kDa
55 kDa
43 kDa
CATB,37kDa
34 kDa
26 kDa
17 kDa
10 kDa

## Slide 4
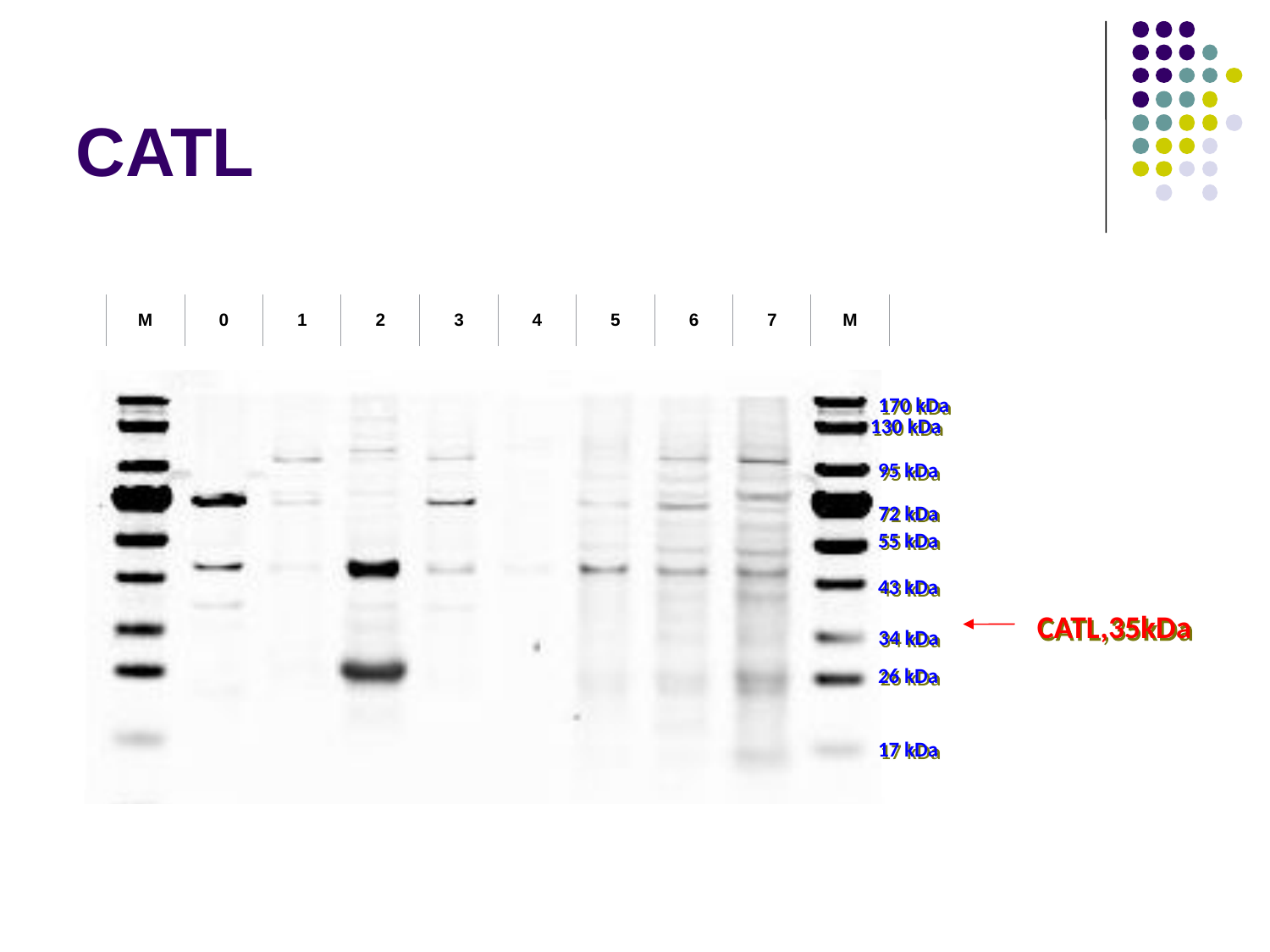

# CATL
| M | 0 | 1 | 2 | 3 | 4 | 5 | 6 | 7 | M |
| --- | --- | --- | --- | --- | --- | --- | --- | --- | --- |
170 kDa
130 kDa
95 kDa
72 kDa
55 kDa
43 kDa
CATL,35kDa
34 kDa
26 kDa
17 kDa

## Slide 5
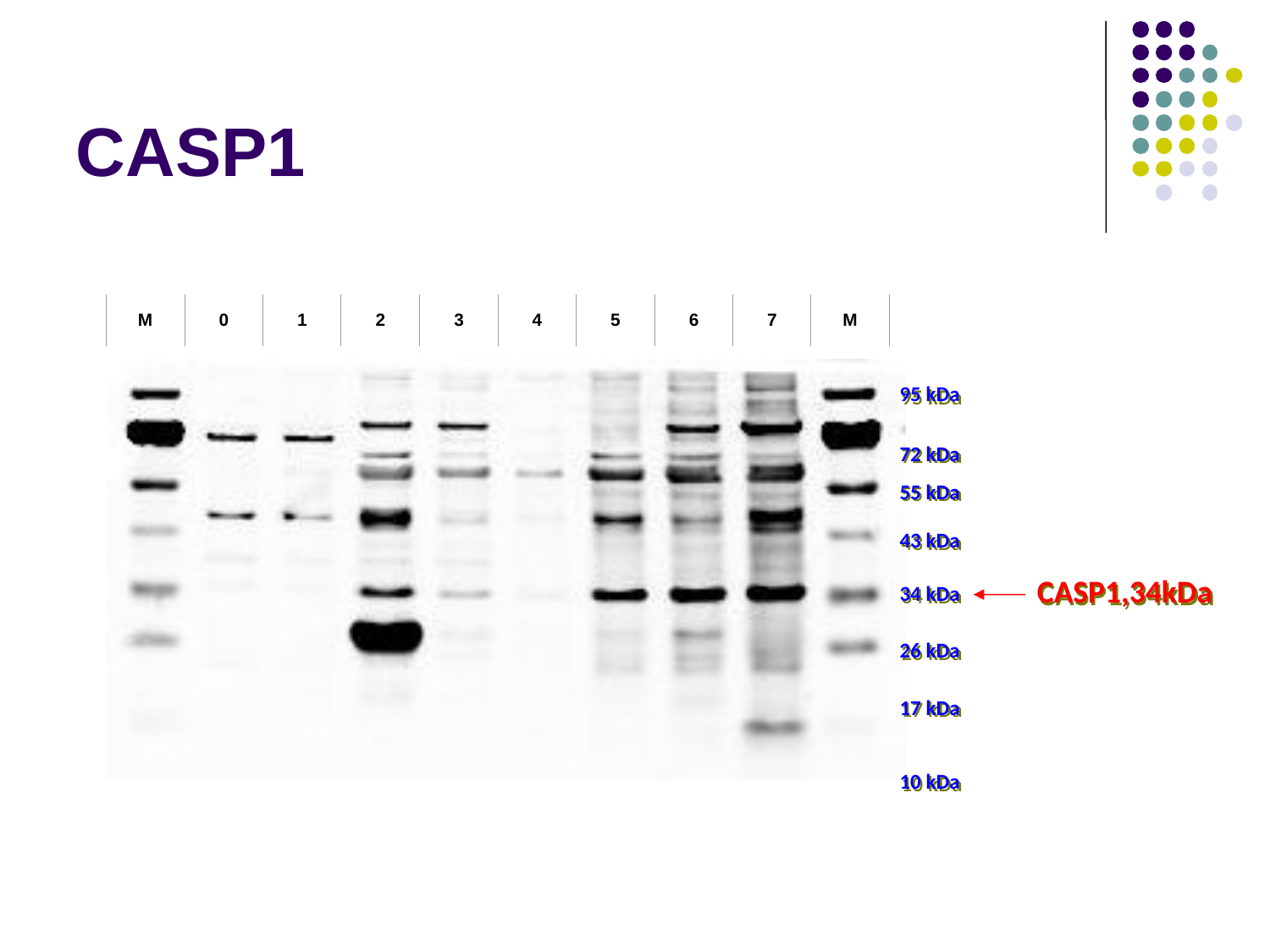

# CASP1
| M | 0 | 1 | 2 | 3 | 4 | 5 | 6 | 7 | M |
| --- | --- | --- | --- | --- | --- | --- | --- | --- | --- |
95 kDa
72 kDa
55 kDa
43 kDa
CASP1,34kDa
34 kDa
26 kDa
17 kDa
10 kDa

## Slide 6
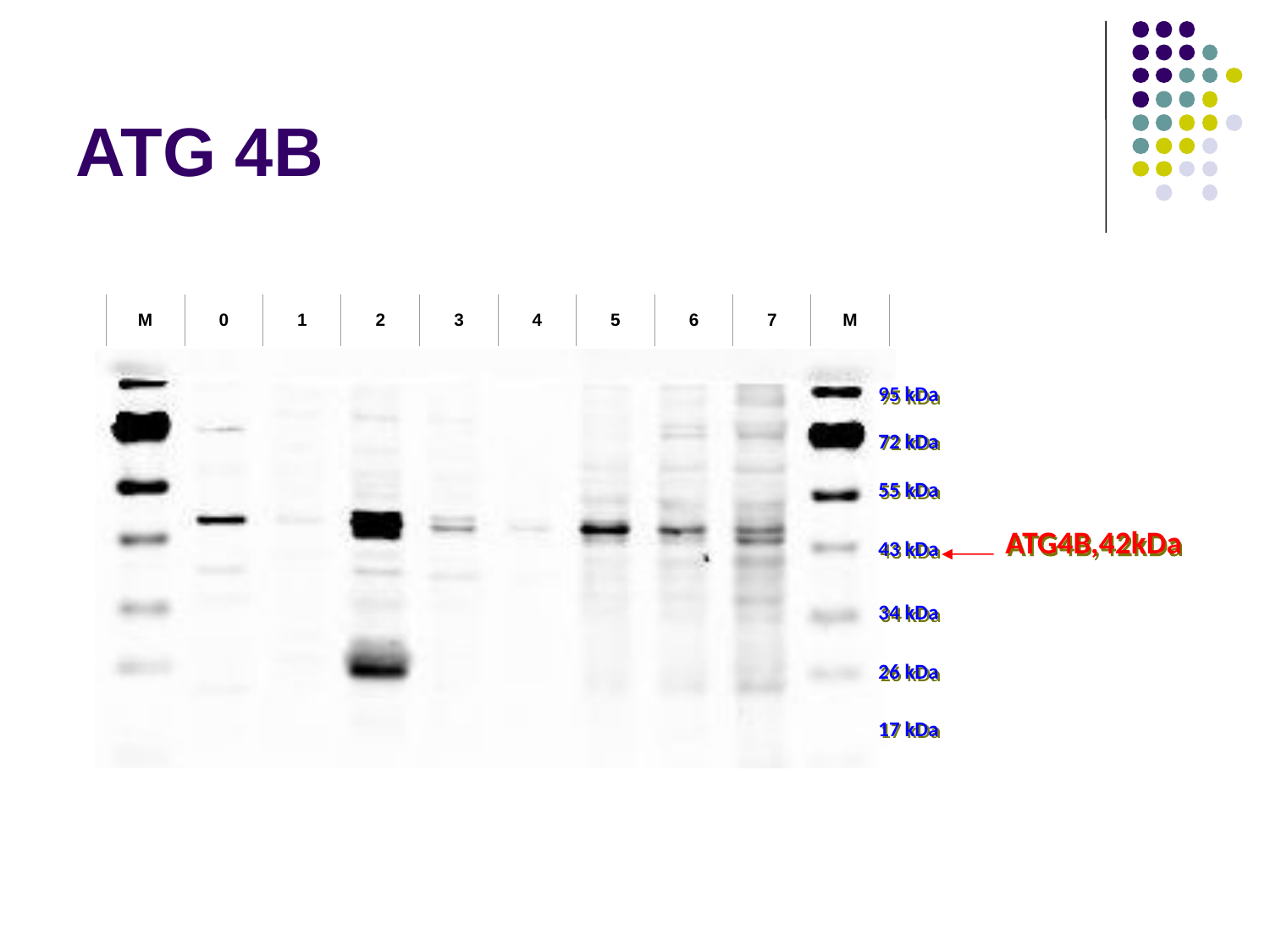

# ATG 4B
| M | 0 | 1 | 2 | 3 | 4 | 5 | 6 | 7 | M |
| --- | --- | --- | --- | --- | --- | --- | --- | --- | --- |
95 kDa
72 kDa
55 kDa
ATG4B,42kDa
43 kDa
34 kDa
26 kDa
17 kDa

## Slide 7
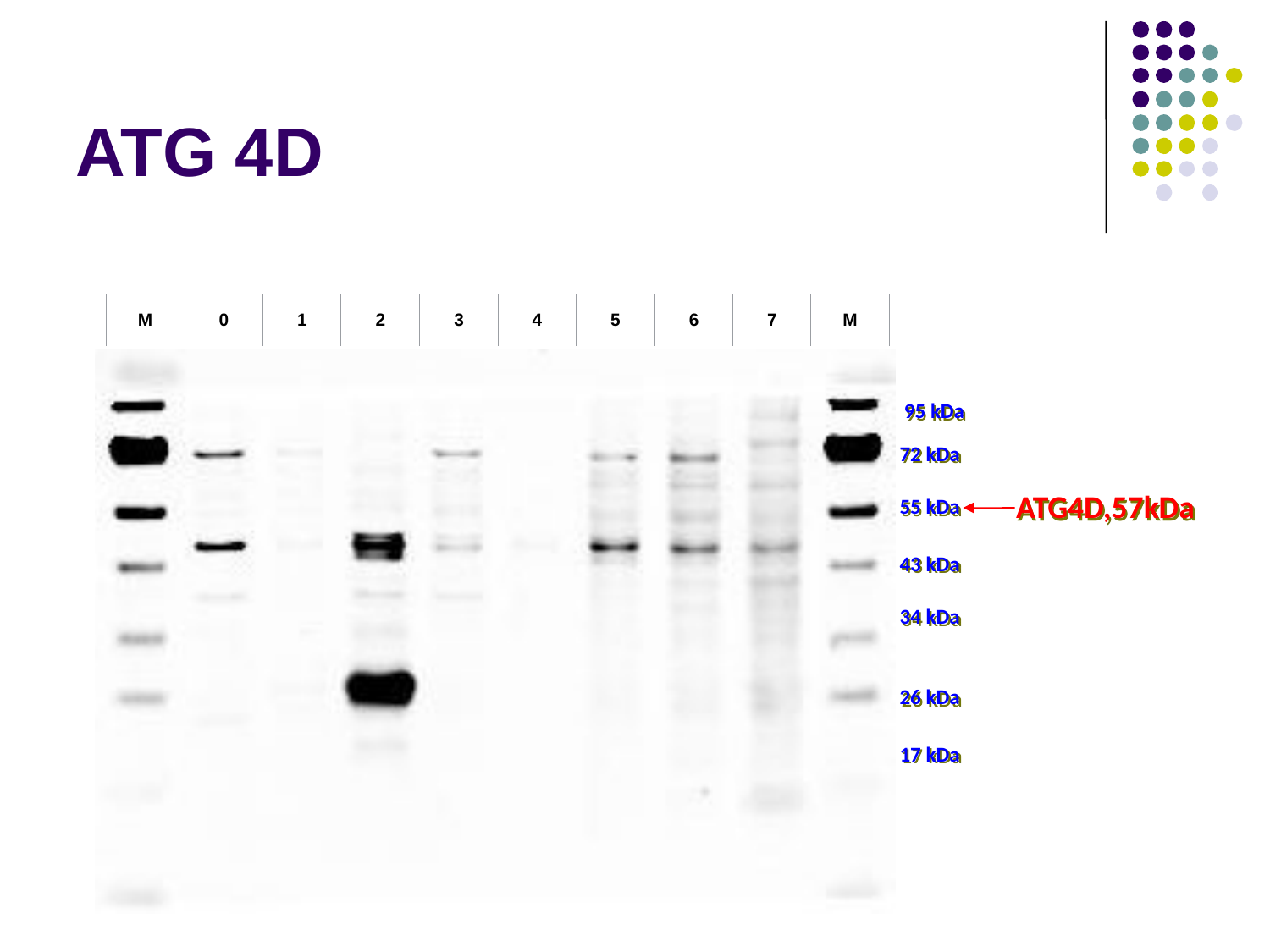

# ATG 4D
| M | 0 | 1 | 2 | 3 | 4 | 5 | 6 | 7 | M |
| --- | --- | --- | --- | --- | --- | --- | --- | --- | --- |
95 kDa
72 kDa
ATG4D,57kDa
55 kDa
43 kDa
34 kDa
26 kDa
17 kDa

## Slide 8
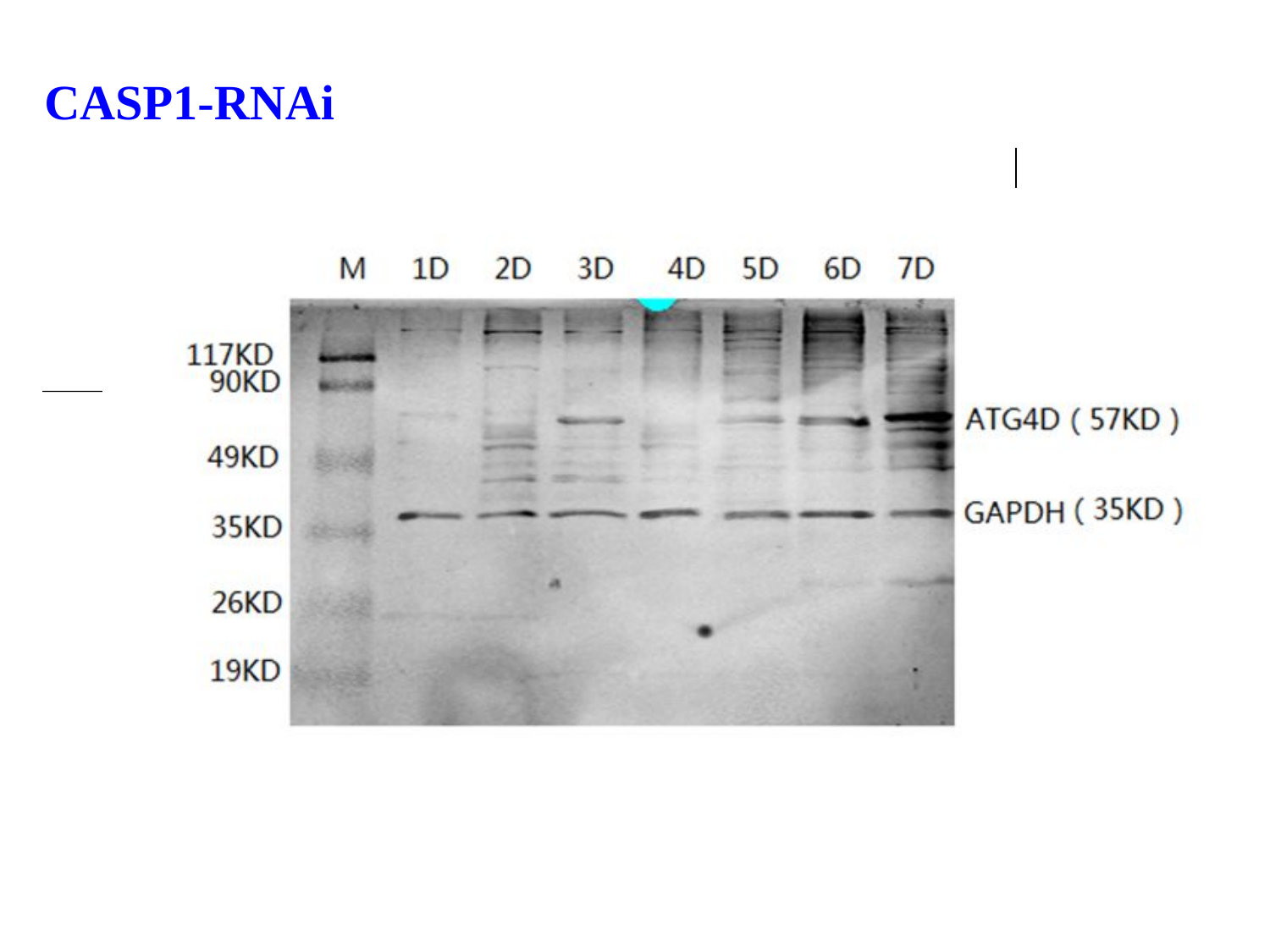

CASP1-RNAi

## Slide 9
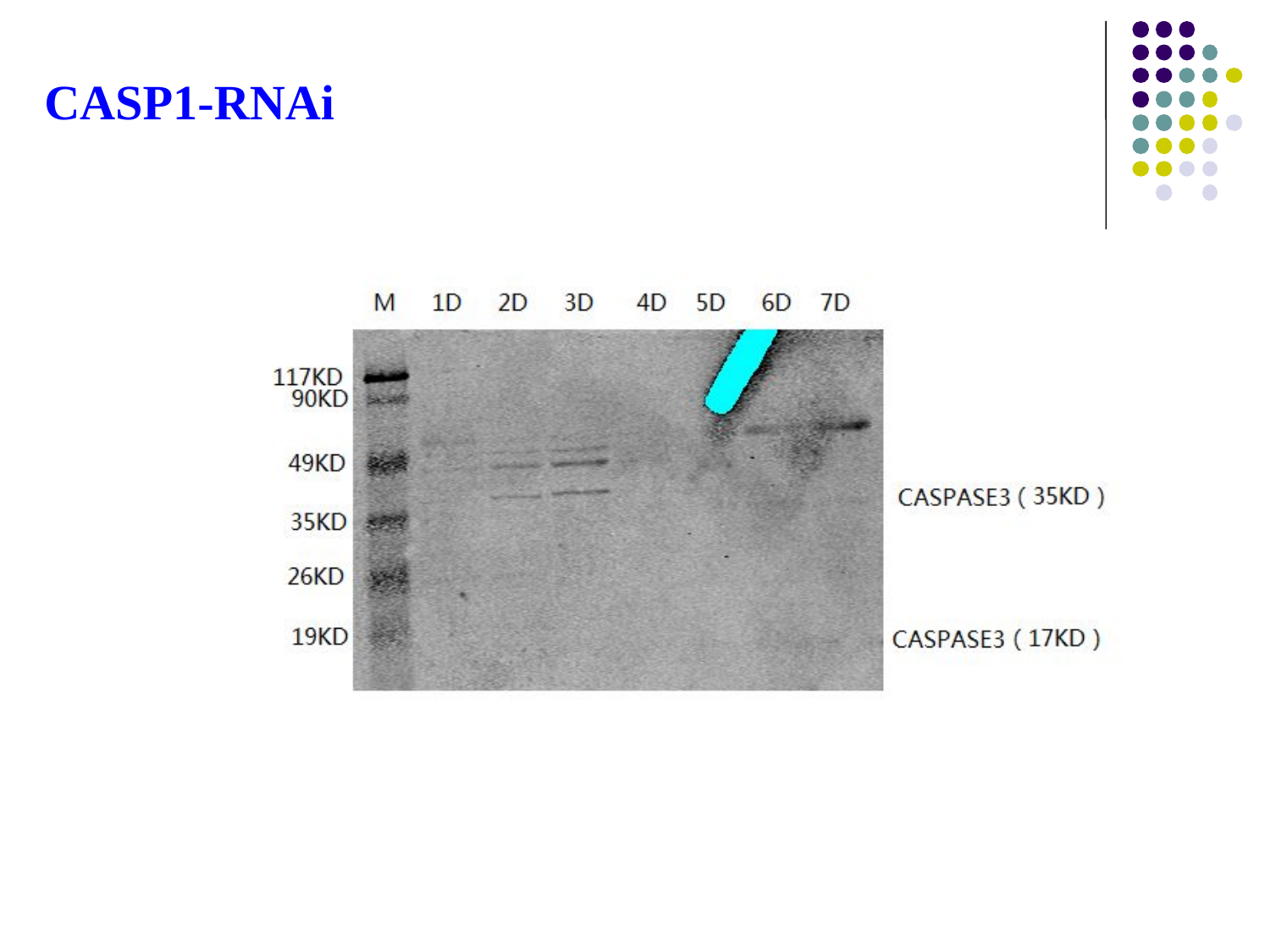

CASP1-RNAi

## Slide 10
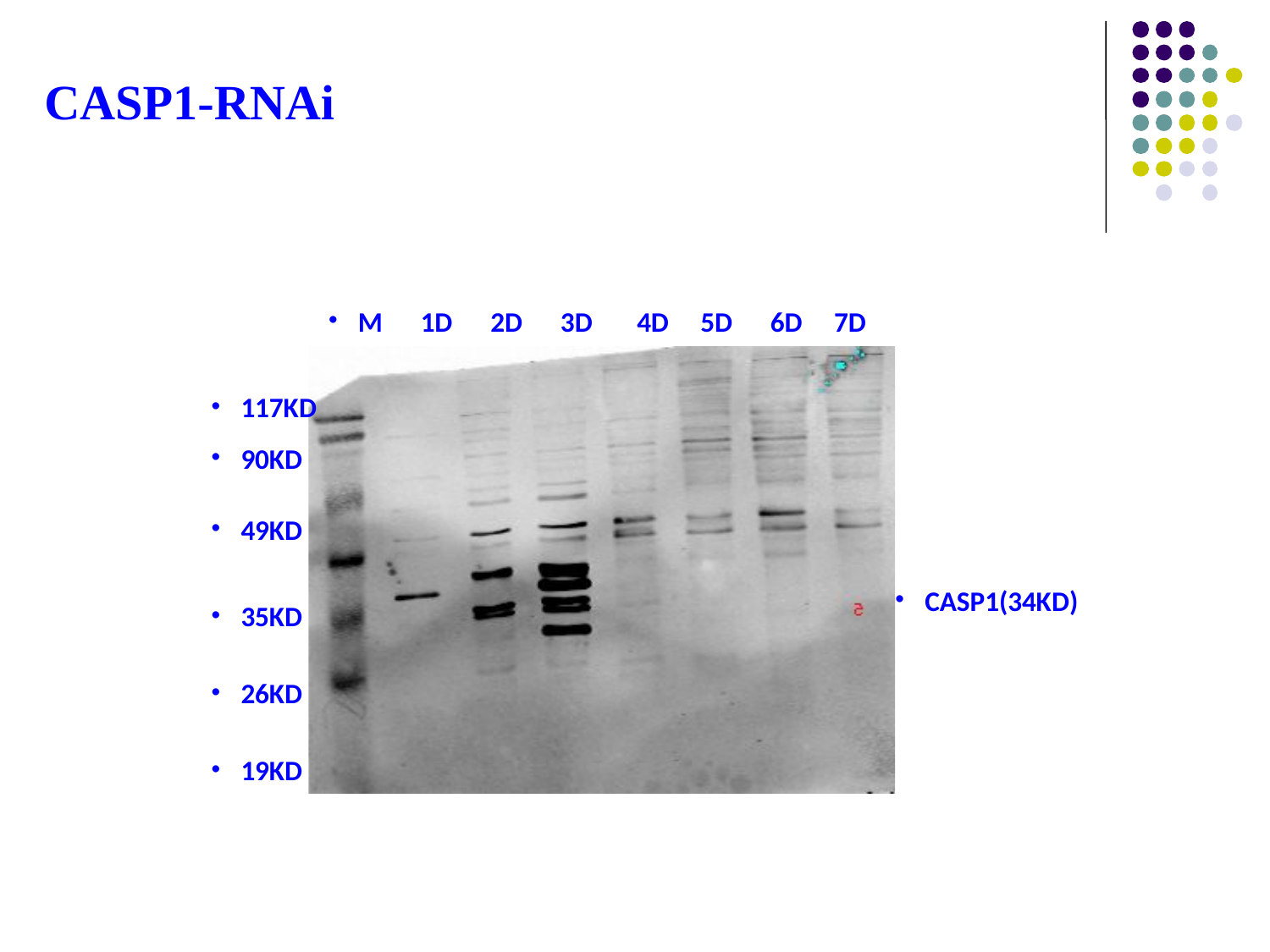

CASP1-RNAi
M 1D 2D 3D 4D 5D 6D 7D
117KD
90KD
49KD
35KD
26KD
19KD
CASP1(34KD)

## Slide 11
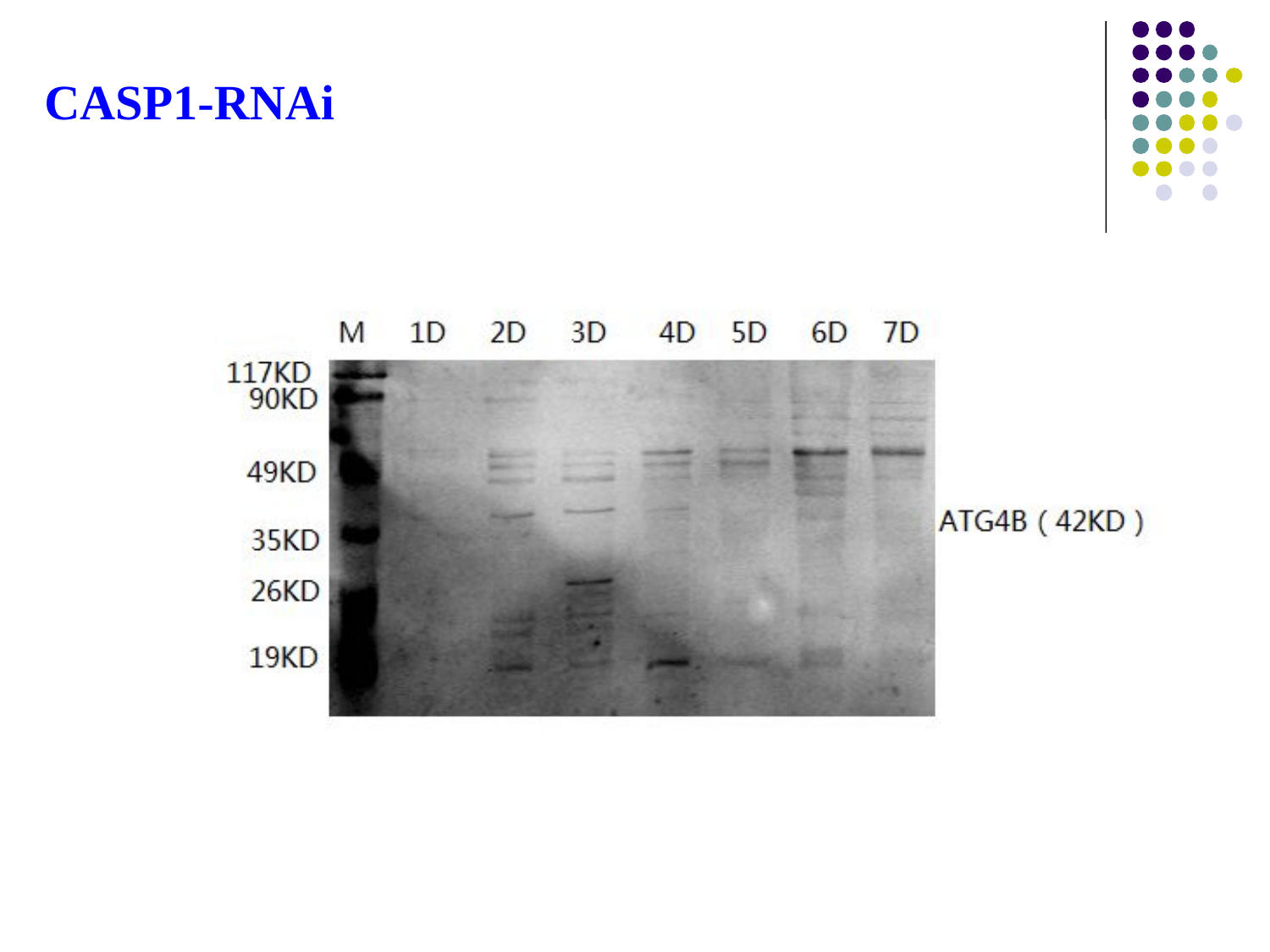

CASP1-RNAi

## Slide 12
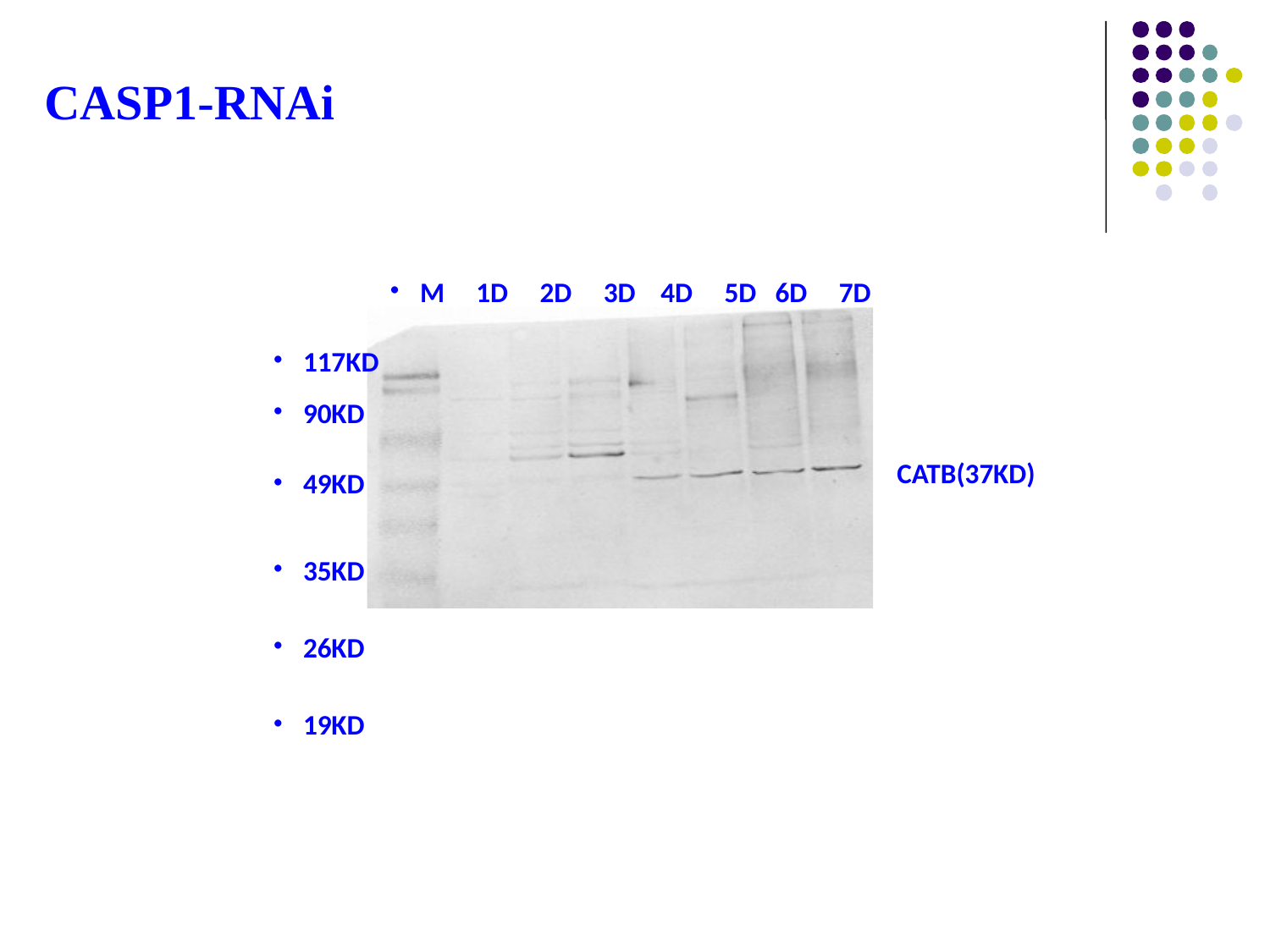

CASP1-RNAi
M 1D 2D 3D 4D 5D 6D 7D
117KD
90KD
49KD
35KD
26KD
19KD
CATB(37KD)

## Slide 13
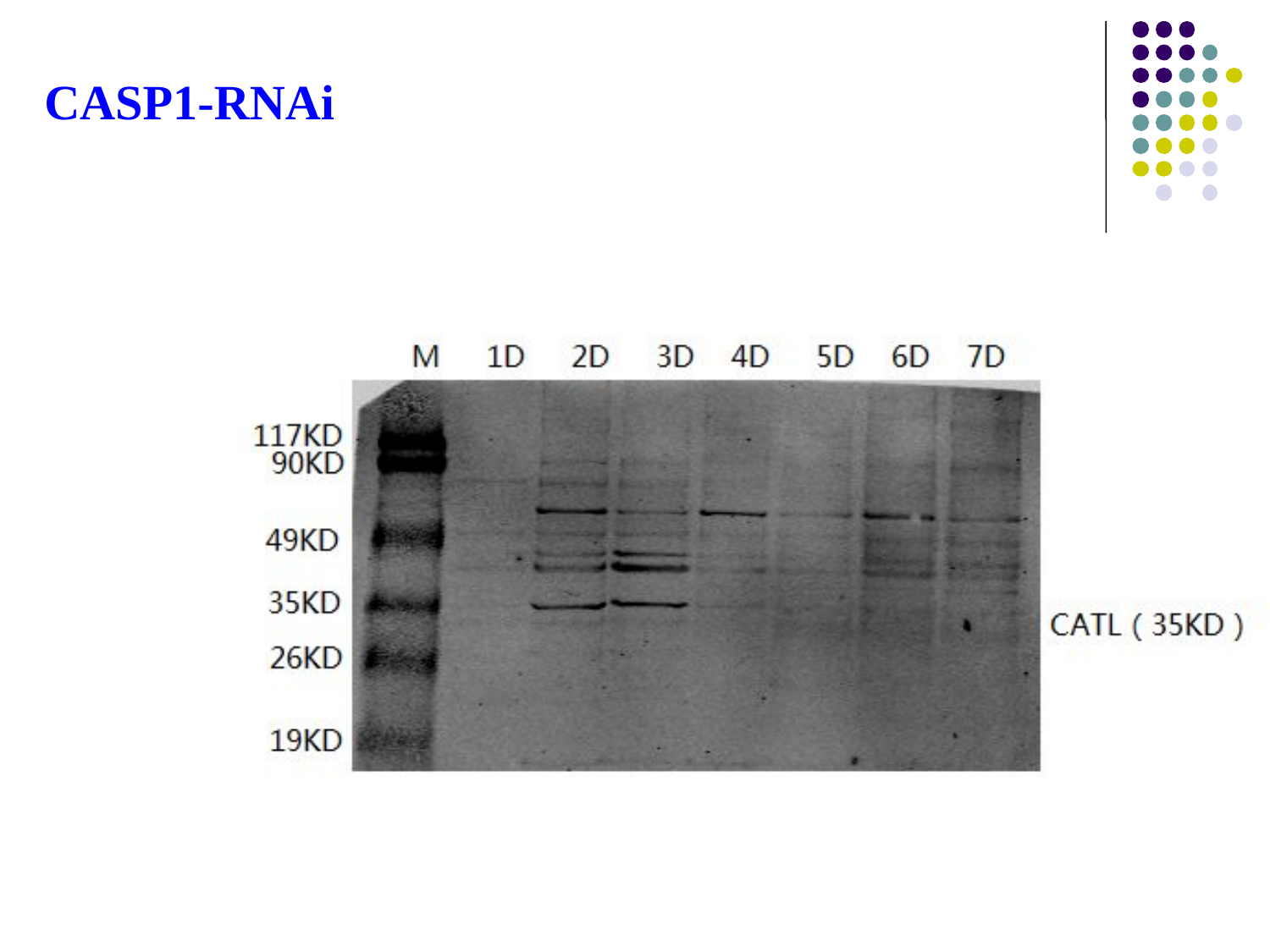

CASP1-RNAi
